# On the optimality of the neighbor-joining algorithm

**DOI:** 10.1186/1748-7188-3-5

**Published:** 2008-04-30

**Authors:** Kord Eickmeyer, Peter Huggins, Lior Pachter, Ruriko Yoshida

**Affiliations:** 1 Department of Computer Science, Humboldt University, Unter den Linden 6, 10099 Berlin, Germany; 2 Department of Mathematics, University of California at Berkeley Berkeley, CA 94720-3840, USA; 3 Department of Statistics, University of Kentucky Lexington, KY 40506, USA

## Abstract

The popular neighbor-joining (NJ) algorithm used in phylogenetics is a greedy algorithm for finding the balanced minimum evolution (BME) tree associated to a dissimilarity map. From this point of view, NJ is "optimal" when the algorithm outputs the tree which minimizes the balanced minimum evolution criterion. We use the fact that the NJ tree topology and the BME tree topology are determined by polyhedral subdivisions of the spaces of dissimilarity maps $R_{+}^{\left(\begin{array}{c} n\\ 2 \end{array}\right)}$ to study the optimality of the neighbor-joining algorithm. In particular, we investigate and compare the polyhedral subdivisions for *n *≤ 8. This requires the measurement of volumes of spherical polytopes in high dimension, which we obtain using a combination of Monte Carlo methods and polyhedral algorithms. Our results include a demonstration that highly unrelated trees can be co-optimal in BME reconstruction, and that NJ regions are not convex. We obtain the *l*_2 _radius for neighbor-joining for *n *= 5 and we conjecture that the ability of the neighbor-joining algorithm to recover the BME tree depends on the diameter of the BME tree.

## 1 Introduction

The popular neighbor-joining algorithm used for phylogenetic tree reconstruction [1] has recently been "revealed" to be a greedy algorithm for finding the balanced minimum evolution tree associated to a dissimilarity map [2]. This means the following:

Let $D={\left\{d_{ij}\right\}}_{i,j=1}^{n}$ be a dissimilarity map (this is an *n *× *n *symmetric matrix with zeroes on the diagonals and non-negative real entries). The *balanced minimum evolution problem *is to find the unrooted binary tree *T *with *n *leaves that minimizes

(1) $$\frac{1}{\left|o(T)\right|}\sum_{\left(x_{1},...,x_{n})\in o(T\right)}\left[\frac{1}{2}\sum_{i=1}^{n}d_{x_{i}x_{i+1}}\right].$$

Here *o*(*T*) is the set of all cyclic permutations of the leaves that arise from planar embeddings of *T *and *x*_*i *_are leaves of *T*. Denote by $p_{ij}^{T}$ the set of internal vertices in a tree *T *on the path between *i *and *j*. Then (1) is equivalent to minimizing

(2) $$\sum_{ij}\lambda_{ij}^{T}d_{ij}$$

where $\lambda_{ij}^{T}=\prod_{v\in p_{ij}^{T}}{\left(deg(v)-1\right)}^{-1}$ if *i *≠ *j *and $\lambda_{ij}^{T}=0$. In [3], Day shows that choosing a minimizing tree for (2) from among the (2*n*-5)!! unrooted binary trees is an *NP*-hard problem. Yet it is desirable to find algorithms for minimizing (2) because of the following statistical interpretation:

### Definition 1.1

*Let T be a tree with n leaves and l*: *E*(*T*) → R*an assignment of lengths to the edges. Then the length l*(*T*) *of T is defined to be*

$$l(T)=\sum_{e\in E(T)}l(e).$$

### Theorem 1.2

([4])*Let T be a binary tree with edge lengths given by l*: *E*(*T*) → R_+ _*and *$D={\left\{d_{ij}\right\}}_{i,j=1}^{n}$*a dissimilarity map. If the variance of d_ij _is proportional to *$2^{\left|p_{ij}^{T}\right|}$*(i. e., var*(*d_ij_*) = $c2^{\left|p_{ij}^{T}\right|}$*for some constant c) then (2) is the minimum variance tree length estimator of T. Moreover, the weighted least squares tree length estimate is equal to (2)*.

This result provides a weighted least squares rationale for the minimization of (2), and highlights the importance of understanding the *balanced minimum evolution polytope*:

### Definition 1.3

The balanced minimum evolution polytope is the convex hull of the vectors

$$\left\{\left[\lambda_{12}^{T},\lambda_{13}^{T},...,\lambda_{ij}^{T},...,\lambda_{n-1,n}^{T}\right]:T\;is\;a\;tree\;with\;n\;leaves\right\}$$

**Example. **There are four trees with *n *= 4 leaves. They are the 3 binary trees and the star-shaped tree. In this case the balanced minimum evolution polytope is the convex hull of the vectors:

$$\begin{array}{ll} \left[\frac{1}{2},\frac{1}{4},\frac{1}{4},\frac{1}{4},\frac{1}{4},\frac{1}{2}\right] & \text{T is the tree with leaves }1,2\text{ seperated from }3,4,\\ \left[\frac{1}{4},\frac{1}{2},\frac{1}{4},\frac{1}{4},\frac{1}{2},\frac{1}{4}\right] & \text{T is the tree with leaves }1,3\text{ seperated from }2,4,\\ \left[\frac{1}{4},\frac{1}{4},\frac{1}{2},\frac{1}{2},\frac{1}{4},\frac{1}{4}\right] & \text{T is the tree with leaves }1,4\text{ seperated from }2,3,\\ \left[\frac{1}{3},\frac{1}{3},\frac{1}{3},\frac{1}{3},\frac{1}{3},\frac{1}{3}\right] & \text{T is the star-shaped tree}. \end{array}$$

The balanced minimum evolution polytope in this case is a triangle in R^6^. Note that the star-shaped tree is in the interior of the triangle.

For any dissimilarity map, the trees which minimize (2) will be vertices of the balanced minimum evolution polytope; these are always the binary trees. In fact, for such trees $\lambda_{ij}^{T}=2^{1-\left|p_{ij}^{T}\right|}$; this is Pauplin's formula [5].

The BME polytope lies in $R^{\left(\begin{array}{c} n\\ 2 \end{array}\right)}$ and has dimension $\left(\begin{array}{c} n\\ 2 \end{array}\right)$ - *n*. The normal fan [6] of the BME polytope gives rise to *BME cones *which form a polyhedral subdivision of the space of dissimilarity maps $R_{+}^{\left(\begin{array}{c} n\\ 2 \end{array}\right)}$. They describe, for each tree *T*, those dissimilarity maps for which *T *minimizes (2). We provide an introduction to the necessary polyhedral combinatorics in Section 2, and discuss the polytope in more detail in Section 3.

The neighbor-joining algorithm is a greedy algorithm for finding an approximate solution to (2). We omit a detailed description of the algorithm here – readers can consult [2] – but we do mention the crucial fact that the selection criterion is linear in the dissimilarity map [7]. Thus, the NJ algorithm will pick pairs of leaves to merge in a particular order and output a particular tree *T *if and only if the pairwise distances satisfy a system of linear inequalities, whose solution set forms a polyhedral cone in $R^{\left(\begin{array}{c} n\\ 2 \end{array}\right)}$. We call such a cone a *neighbor-joining cone*. or *NJ cone*. The NJ algorithm will output a particular tree *T *if and only if the distance data lies in a union of NJ cones. In Section 4 we show that the NJ cones partition $R^{\left(\begin{array}{c} n\\ 2 \end{array}\right)}$, but do not form a fan. This has important implications for the behavior of the NJ algorithm.

Our main result is a comparison of the neighbor-joining cones with the normal fan of the balanced minimum evolution polytope. This means that we characterize those dissimilarity maps for which neighbor-joining, despite being a greedy algorithm, is able to identify the balanced minimum evolution tree. These results are discussed in Section 5.

## 2 Polyhedral preliminaries

In this section we will introduce some of the elementary polyhedral combinatorics necessary for this paper. For more details see [8].

Let {*y*_1_, *y*_2_*, ..., y*_*m*_} be a finite set of points in R^*d*^. An *affine linear combination *is a linear combination of the form

$$\begin{array}{ccc} y=\sum_{i=1}^{m}\alpha_{i}y_{i}, & \text{where} & \sum_{i=1}^{m}\alpha_{i}=1. \end{array}$$

A *convex linear combination *is an affine linear combination with nonnegative linear coefficients, i.e. *α*_*i *_≥ 0 for *i *= 1, ..., *m*. The *affine hull *of a set *C *⊆ R^*d *^is the set of all affine linear combinations of vectors from *C*. The *convex hull *of *C *is the set of all convex linear combinations on vectors from *C*. A set is called *affinely closed *or an *affine space *if it equals its affine hull, and it is called *convex *if it equals its convex hull. Every affine space *A *⊂ R^*d *^can be written as

*a *+ *V *= {*a *+ *v *: *v *⊆ *V*}

where *V *⊆ R^*d *^is a subspace and *a *∈ *A*. *V *is uniquely determined by *A *and the *affine dimension *of *A *is defined to be the dimension of *V*.

Given two distinct points *x*, *y *∈ R^*d*^, the set [*x, y*] = {*αx *+ (1 - *α*)*y *: 0 ≤ *α *≤ 1} of all convex combinations of *x *and *y *is called the *interval *with endpoints *x *and *y*. Then *C *⊂ R^*d *^is convex iff [*x, y*] ⊂ *C *for any two *x, y *∈ *C*.

**Figure 1:**
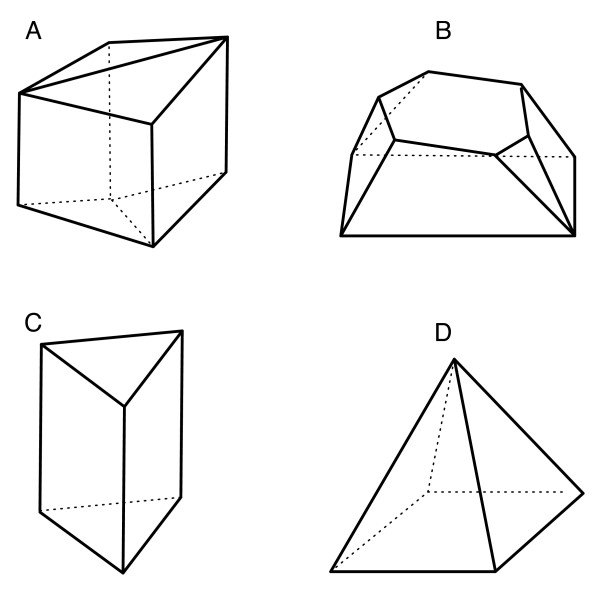
The four types of facets of *P*.

Let *A*_1_, *A*_2_, ..., *A*_*N *_∈ R^*d *^and let *b*_1_, *b*_2_, ..., *b*_*N *_∈ R. Then the set

$$P:=\{x\in R^{d}:A_{i}\cdot x\leq b_{i}\text{ for }i=1,2,...,N\}$$

is called a *polyhedron*. The convex hull of a finite set of points in R^*d *^is called a *polytope *and the Weyl-Minkowski Theorem says that a polytope is a bounded polyhedron [9]. Polytopes are familiar objects in geometry. In the plane, polytopes are precisely the convex polygons. In R^3^, examples of polytopes are shown in Figure 1. The dimension dim *P *of a polytope or polyhedron *P *is defined to be the dimension of the affine hull of *P*.

A (*d *- 1) dimensional affine set in R^*d *^is called a *hyperplane *and every hyperplane can be represented as {*x *∈ R^*d*^: *n*·*x *= *b*} for some *n *≠ 0 ∈ R^*d *^and *b *∈ R, where *n*·*x *is the dot-product of *n *and *x*. We call *n *a *normal vector *of this hyperplane.

Let *H *:= {*x *∈ R^*d *^: *h*·*x *≤ *b*}, where *h *≠ 0 ∈ R^*d *^and *b *∈ R, be an *affine half space*. Then if *P *⊂ *H *and *P *⋂ {*x *∈ R^*d *^: *h*·*x *= *b*} ≠ ∅, then *H *is called a *supporting hyperplane *of *P*. A subset *F *of *P *is called a *face *if *F *= *P *or *F *= *P *⋂ *H*, where *H *is a supporting hyperplane. Faces of polyhedra are polyhedra and faces of polytopes are polytopes.

Faces of dimension 0 are called *vertices*, faces of dimension 1 are called *edges*, and faces of dimension *d *- 1 are called *facets*. The *f-vector *of *P *is the vector (*f*_0_, *f*_1_, *f*_2_, ...), where *f*_*i *_is the number of faces of dimension *i *of *P'*. For example, consider the 3-dimensional polytope labeled 'C' in Figure 1. This polytope has 6 vertices, 9 edges, and 5 facets (3 quadrilaterals and 2 triangles), and so its *f*-vector is (6, 9, 5).

A polyhedron *C *is a *cone *if it can be written as

$$C=\left\{\sum_{i=1}^{N}\alpha_{i}y_{i}:\alpha_{i}\geq 0\text{ for }i=1,...,N\right\}$$

for some *y*_1_, ..., *y*_*N *_∈ R^*d*^. This is equivalent to the existence of a matrix *A *∈ R^*m *× *n *^such that *C *= {*x *: *A*_*x *_≥ 0}. A cone is *pointed *if its lineality space is {0}.

Given a face *F *of a polytope P, the *normal cone N*(*F*) is the set of all vectors *c *for which *c*·*v *= max_*x*∈*P *_*c*·*x *for all *v *∈ *F*. The collection of relative interiors of normal cones of faces of *P *partition R^*d*^, and for each face we have dim(*F*) + dim(*N*(*F*)) = *d*. The collection of normal cones of faces of *P *is called the *normal fan *of *P*.

Given a polyhedron *P*, the *lineality space *of *P *is the set of vectors *v *for which *y *+ *c·v *∈ *P *for all *y *∈ *P *and *c *∈ *R*. The largest such subspace is called *lineality space *of *P*. If a polyhedron *P *has lineality space *V*, we can let *V' *be the orthogonal complement *V' *(i.e. *V *⊕ *V' *= R^*d*^) and consider the polyhedron *P' *:= *P *⋂ *V'*, which has lineality space {0}.

## 3 The balanced minimum evolution polytope

Throughout this paper we work with binary unrooted trees on *n *leaves labeled {1, ..., *n*}. Such trees are also known as *phylogenetic X-trees*. We refer the reader to [10] for more detail about such trees, and for related definitions. Recall there are 2*n *- 3 edges in an unrooted tree with *n *leaves. For a fixed tree topology *T*, let *B*_*T *_be the $\left(\begin{array}{c} n\\ 2 \end{array}\right)$ × (2*n *- 3) matrix with rows indexed by pairs of leaves and columns indexed by edges in *T *defined as follows:

$$B_{T}(\{a,b\},e)=\{\begin{array}{cc} 1 & \text{if edge }e\text{ is in the path from leaf }a\text{ to leaf }b,\\ 0 & \text{otherwise}. \end{array}$$

**Figure 2:**
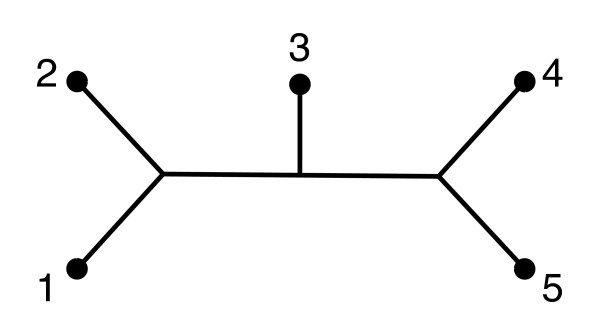
A tree with five leaves.

For example, for the tree in Figure 2,

$$B_{T}=\left(\begin{array}{ccccccc} 1 & 1 & 0 & 0 & 0 & 0 & 0\\ 1 & 0 & 1 & 0 & 0 & 1 & 0\\ 0 & 1 & 1 & 0 & 0 & 1 & 0\\ 1 & 0 & 0 & 1 & 0 & 1 & 1\\ 0 & 1 & 0 & 1 & 0 & 1 & 1\\ 0 & 0 & 1 & 1 & 0 & 0 & 1\\ 1 & 0 & 0 & 0 & 1 & 1 & 1\\ 0 & 1 & 0 & 0 & 1 & 1 & 1\\ 0 & 0 & 1 & 0 & 1 & 0 & 1\\ 0 & 0 & 0 & 1 & 1 & 0 & 0 \end{array}\right)$$

where its rows are indexed by pairs of leaves (1, 2), (1, 3), (2, 3), (1, 4), (2, 4), (3, 4), (1, 5), (2, 5), (3, 5), (4, 5) and its columns are indexed by edges (1, *a*), (2, *a*), (3, *b*), (4, *c*), (5, *c*), (*a*, *b*), (*b*, *c*) with *a *is an internal node adjacent to leaves 1 and 2, *c *is an internal node adjacent to leaves 4, 5, and *b *is an internal node adjacent to nodes 3, *a *and *c*. Given edge lengths *l *: *E*(*T*) → R_+ _we let **b **be the vector with components *l*(*e*) as *e *ranges over *E*(*T*). Any dissimilarity map **d **(encoded as a row vector) can now be written as

**d **= *B*_*T *_**b **+ **e**

where **e **is a vector of "error" terms that are zero when **d **is a tree metric.

The weighted least squares solution for the edge lengths **b **assuming a variance matrix *V *with off-diagonal entries $v_{ij}=\lambda_{ij}^{T}$ (as defined in the introduction) and dissimilarity map **d **is given by

$$\hat{b}={\left(B_{T}^{t}V^{-1}B_{T}\right)}^{-1}B_{T}^{t}V^{-1}d,$$

where ·^*t *^denotes matrix transpose. The length of *T *with respect to the least squares edge lengths is then

*l*(*T*) = **v**_*T*_·**d**,

where $v_{T}=V^{-1}B_{T}{\left(B_{T}^{t}V^{-1}B_{T}\right)}^{-1}$**1 **and **1 **is the vector of all 1's. We call the vectors **v**_*T *_the balanced minimum evolution vectors (or BME vectors). In the case of Figure 2, the BME vector is

$$v_{T}=\left[\frac{1}{2},\frac{1}{4},\frac{1}{4},\frac{1}{4},\frac{1}{4},\frac{1}{4},\frac{1}{4},\frac{1}{4},\frac{1}{4},\frac{1}{2}\right].$$

The BME method is equivalent to minimizing the linear functional **v**_*T*_·**d **over all BME vectors for all tree topologies *T*. The BME polytope is the convex hull of all BME vectors in $R^{\left(\begin{array}{c} n\\ 2 \end{array}\right)}$. The following facts follow from the definition of the balanced minimum evolution tree:

### Lemma 3.1

*The vertices of the BME polytope are the BME vectors of binary trees. The BME vector of the star phylogeny lies in the interior of the BME polytope, and all other BME vectors lie on the boundary of the BME polytope*.

The normal fan of a BME polytope partitions the space $R^{\left(\begin{array}{c} n\\ 2 \end{array}\right)}$ of dissimilarity maps into cones, one for each tree. We call these *BME cones*. They completely characterize the BME method: *T *is the BME tree topology if and only if the dissimilarity map *D *lies in the BME cone of *T*.

For a leaf node *a *in a binary unrooted tree, the *shift vector ***s**_*a *_is the dissimilarity map in which *a *is at distance 1 from all other leaves, and all other distances are 0 (see [11] for the description of shift vectors). According to [5], for a tree *T*, (**v**_*T*_)_*ab *_gives the probability that *a *will immediately precede *b *in a random circular ordering of *T*. Thus the dot-product of a BME vector with a shift vector must necessarily equal 1, and in fact the lineality space of BME cones is spanned by shift vectors. So when we describe a BME cone we will always describe just the pointed component, i.e. modulo the lineality space of shift vectors.

As part of our computational study, we computed the BME polytope and BME cones for trees with *n *= 4, 5, 6, 7, 8 leaves using the software polymake [12]. In Table 1 we display some of the components of *f*-vectors we were able to compute. This provides information about the polytopes: Recall that the *i*th component of the *f*-vector of a polytope is the number of faces of dimension *i *- 1. For example, the first component in each vector in Table 1 is the number of 0-dimensional faces (vertices) of the corresponding BME polytope, i.e., the number of binary trees.

**Table 1:** The *f*-vector for small BME polytopes.

#leaves	dim(BME polytope)	*f*-vector
4	2	(3,3)
5	5	(15, 105, 250, 210, 52)
6	9	(105, 5460, ?, ?, ?, 90262)
7	14	(945, 445410, ?, ?, ?, ?, ?)
ℬ	ℬ	ℬ
*n*	$\left(\begin{array}{c} n\\ 2 \end{array}\right)$ - *n*	((2*n *- 5)!!,?, ...)

**Figure 3:**
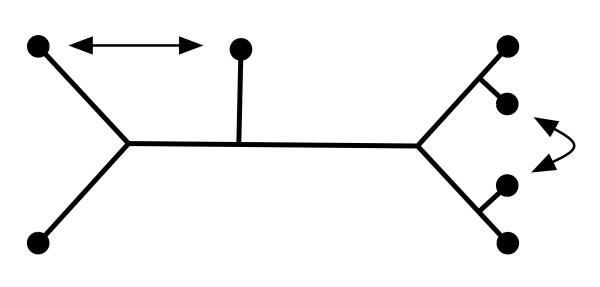
**The non-edges on the BME polytope for *n *= 7. **Two trees will form a non-edge if and only if they are trees that have three cherries, and differ by the pair of leaf exchanges shown in the figure. There are two ways to perform each leaf-exchange, so each binary tree with three cherries is not adjacent to 4 trees.

We found that the edge graph of the BME polytope is the complete graph for *n *= 4, 5, 6 which means that for every pair of trees *T*_1 _and *T*_2 _with the same number (≤ 6) of leaves, there is a dissimilarity map for which *T*_1 _and *T*_2 _are (the only) co-optimal BME trees. However, for *n *= 7, the BME polytope does in fact have one combinatorial type of non-edge. Namely, two bifurcating trees with seven leaves and three cherries (two leaves adjacent to the same node in the tree) will form a non-edge if and only if they are related by two leaf exchanges as depicted in Figure 3. This completely characterizes the non-edges for *n *= 7. It is an interesting open problem to characterize the non-edges of the BME polytope in general.

## 4 Neighbor-joining cones

The neighbor-joining algorithm takes as input a dissimilarity map and outputs a tree. The tree is constructed "one cherry at a time". In each step the algorithm chooses a pair of leaves *a *and *b *that minimize the *Q-criterion*, which is defined by the formula

(3) $$q_{ab}:=(n-2)d_{ab}-\sum_{k=1}^{N}d_{ak}-\sum_{k=1}^{n}d_{kb}.$$

The nodes *a, b *are replaced by a single node *z*, and new distances *d*_*zk *_are obtained by a straightforward linear combination of the original pairwise distances:$d_{zk}:=\frac{1}{2}(d_{ak}+d_{bk}-d_{ab})$. Then the NJ method is applied recursively.

We note that since new distances *d*_*zk *_are always linear combinations of the previous distances, all Q-criteria computed throughout the NJ algorithm are linear combinations of the original pairwise distances. Thus, for a fixed *n*, for every possible ordering *σ *of picked cherries that results in one of the trees *T *with *n *leaves there is a polyhedral cone *C*_*σ *_⊂ $R^{\left(\begin{array}{c} n\\ 2 \end{array}\right)}$ of dissimilarity maps. The set of all neighbor-joining cones is denoted by $C_{n}$. Their union $\cup_{C\in C_{n}}C$ is all of of $R^{\left(\begin{array}{c} n\\ 2 \end{array}\right)}$, and the intersection of any two cones is a subset – but not necessarily a face – of the boundary of each of the cones. Given an input from the interior of *C*_*σ*_, the NJ algorithm will pick the cherries in the order *σ *and output the corresponding tree. For inputs **d **on the boundary of one (and therefore at least two) of the cones, the order in which NJ picks cherries is undefined, because at some point there will be two cherries both of which have minimal Q-criterion. We call the cones *C*_*σ *_*neighbor-joining cones*, or *NJ cones*. See [11] for the hyperplane representation of NJ cones and descriptions how to construct each cone.

**Example. **There is only one unlabeled binary tree with 5 leaves and there are 15 distinct labeled trees. For each labeled tree, there are two ways in which a cherry might be picked by the NJ algorithm in the first step. For instance, neighbor-joining applied to any dissimilarity map in *C*_12,45 _or *C*_45,12 _will produce the tree in Figure 2. There are a total of 30 NJ cones for *n *= 5.

We note that all Q-criteria for shift vectors equal -2, so adding any linear combination of shift vectors to a dissimilarity map does not change the relative values of the Q-criteria. Also, after picking a cherry, the reduced distance matrix of a shift vector is again a shift vector. Thus, for any input vector **d**, the behavior of the NJ algorithm on **d **will be the same as on **d **+ **s **if **s **is any linear combination of shift vectors. In fact it can be shown that the lineality space of NJ cones is spanned by shift vectors, just as for BME cones [11]. So from now on, when we refer to NJ cones, we will mean the pointed portion of the cone, i.e. modulo the lineality space.

### Theorem 4.1

*The cones in *$C_{n}$*do not form a fan. In particular, they are not the normal fan of any polytope for n *≥ 5.

The theorem follows from that fact that the NJ cones have rays which are on the boundary of other cones but not rays of them. Thus there are pairs of cones whose intersection is not a face of both cones. We describe the case *n *= 5 in detail; it also suffices to prove the theorem.

We begin by noting that all of the NJ cones are equivalent under the action of the symmetric group on five elements (*S*_5_), where an element of *S*_5 _permutes the five taxa or, equivalently, the rows and columns of the input distance matrix. Each NJ cone is defined by $\left(\left(\begin{array}{c} 5\\ 2 \end{array}\right)-1\right)+\left(\left(\begin{array}{c} 4\\ 2 \end{array}\right)-1\right)=14$ inequalities that are implied by the Q-criteria as the NJ algorithm picks the two cherries. The cones are 5-dimensional, and their intersection with a suitable hyperplane leaves a four dimensional polytope *P*. The *f*-vector of *P *is (14, 32, 27, 9).

The 30 cones share many of their rays, giving a total of 82 rays which decompose into three orbits under the action of *S*_5_. We refer to the types of rays as Type I, Type II and Type III. Each cone has 6 rays of type I, 4 rays of type II and 4 rays of type III. Each ray of type I is the common ray of 3 cones, and belongs to 2 other cones of which it is not a ray (i.e. it is in the interior of a face). Note that this implies that the cones cannot form a fan. The type II rays are contained in 10 cones each, and the type III rays in 12. Type II and III rays are rays of all cones which contain them. For the cone *C*_23,45_, this information is tabulated in Table 2.

**Table 2:** The 14 rays of the cone *C*_23,45_. Each ray is determined by a vector shown in the second column. The third column shows, for each ray, which cones it belongs to. If a cone is starred then the ray is on the boundary of that cone, but not a ray of it.

Type	rays	Cones
I	(-3, 5, -3, -1, 5, -3, -1, 1, 1, -1) (-3, 5, -3, -1, 1, 1, -1, 5, -3, -1) (5, -3, -3, -1, -3, 5, -1, 1, 1, -1) (1, 1, -3, -1, -3, 5, -1, 5, -3, -1) (5, -3, -3, -1, 1, 1, -1, -3, 5, -1) (1, 1, -3, -1, 5, -3, -1, -3, 5, -1)	*C*_23,45_, *C*_23,15_, *C*_23,14_, $C_{12,34}^{*}$, $C_{34,12}^{*}$ *C*_23,45_, *C*_23,15_, *C*_23,14_, $C_{12,35}^{*}$, $C_{35,12}^{*}$ *C*_23,45_, *C*_23,15_, *C*_23,14_, $C_{24,13}^{*}$, $C_{13,24}^{*}$ *C*_23,45_, *C*_23,15_, *C*_23,14_, $C_{25,13}^{*}$, $C_{25,13}^{*}$ *C*_23,45_, *C*_23,15_, *C*_23,14_, $C_{24,35}^{*}$, $C_{35,24}^{*}$ *C*_23,45_, *C*_23,15_, *C*_23,14_, $C_{25,34}^{*}$, $C_{25,34}^{*}$

II	(-1, 1, -1, 1, 1, -1, -1, 1, 1, -1) (-1, 1, -1, -1, 1, 1, 1, 1, -1, -1) (1, 1, -1, -1, -1, 1, -1, 1, -1, 1) (1, -1, -1, 1, -1, 1, -1, 1, 1, -1)	*C*_12,45_, *C*_12,34_, *C*_23,45_, *C*_23,15_, *C*_34,15_, *C*_34,12_, *C*_45,23_, *C*_45,12_, *C*_15,34_, *C*_15,23_ *C*_12,45_, *C*_12,35_, *C*_23,45_, *C*_23,14_, *C*_35,14_, *C*_35,12_, *C*_45,23_, *C*_45,12_, *C*_14,35_, *C*_14,23_ *C*_25,14_, *C*_25,13_, *C*_23,14_, *C*_23,45_, *C*_13,45_, *C*_13,25_, *C*_14,23_, *C*_14,25_, *C*_45,13_, *C*_45,23_ *C*_24,15_, *C*_24,13_, *C*_23,15_, *C*_23,45_, *C*_13,45_, *C*_13,24_, *C*_15,23_, *C*_15,24_, *C*_45,13_, *C*_45,23_

III	(1, -1, -1, 1, 1, -1, -1, -1, 3, -1) (1, -1, -1, -1, -1, 3, 1, 1, -1, -1) (1, -1, -1, 1, 1, -1, -1, -1, 3, -1) (1, -1, -1, -1, -1, 3, 1, 1, -1, -1)	*C*_23,45_, *C*_23,15_, *C*_12,45_, *C*_12,35_, *C*_24,15_, *C*_24,35_, *C*_35,24_, *C*_35,12_, *C*_15,24_, *C*_15,23_, *C*_45,12_, *C*_45,23_ *C*_23,45_, *C*_23,14_, *C*_12,45_, *C*_12,34_, *C*_25,14_, *C*_25,34_, *C*_34,25_, *C*_34,12_, *C*_14,25_, *C*_14,23_, *C*_45,12_, *C*_45,23_ *C*_23,45_, *C*_23,15_, *C*_13,45_, *C*_13,25_, *C*_34,15_, *C*_34,25_, *C*_25,34_, *C*_25,13_, *C*_15,34_, *C*_15,23_, *C*_45,13_, *C*_45,23_ *C*_23,45_, *C*_23,14_, *C*_13,45_, *C*_13,24_, *C*_35,14_, *C*_35,24_, *C*_24,35_, *C*_24,13_, *C*_14,35_, *C*_14,23_, *C*_45,13_, *C*_45,23_

We note that the rays of NJ cones are minimal intersections of NJ cones, and thus give dissimilarity maps for which the NJ algorithm is least stable.

**Example. **Consider two alignments of 5 sequences that are to be used to construct a tree. These may consist of two different genes and for each of them the homologs among 5 genomes. Suppose that distances are estimated using the Jukes-Cantor correction [6,13] separately for each set of sequences. That is, for the first set of sequences

$${\left(D_{1}\right)}_{ij}=-\frac{3}{4}\log(1-\frac{4}{3}f_{ij})$$

where *f*_*ij *_is the fraction of different nucleotides between sequences *i *and *j *in the first set and for the second set

$${\left(D_{2}\right)}_{ij}=-\frac{3}{4}\log(1-\frac{4}{3}g_{ij})$$

where *g*_*ij *_is the fraction of different nucleotides between sequences *i *and *j *in the second set.

If the fractions *f*_*ij *_and *g*_*ij *_are given by

$$\begin{array}{c} f:=\left(\begin{array}{ccccc} 0 & 0.054187 & 0.151108 & 0.368136 & 0.054198\\ 0.054187 & 0 & 0.151117 & 0.054198 & 0.36813\\ 0.151108 & 0.151117 & 0 & 0.054187 & 0.054198\\ 0.368136 & 0.054198 & 0.054187 & 0 & 0.151108\\ 0.054198 & 0.36813 & 0.054198 & 0.151108 & 0 \end{array}\right)\text{ and}\\ g:=\left(\begin{array}{ccccc} 0 & 0.151068 & 0.05414 & 0.368161 & 0.104517\\ 0.151068 & 0 & 0.054245 & 0.054245 & 0.395699\\ 0.05414 & 0.054245 & 0 & 0.151068 & 0.194428\\ 0.368161 & 0.054245 & 0.151068 & 0 & 0.104421\\ 0.104517 & 0.395699 & 0.194428 & 0.104421 & 0 \end{array}\right) \end{array}$$

then we obtain

$$\begin{array}{c} D_{1}=\left(\begin{array}{ccccc} 0 & 0.056244 & 0.168744 & 0.506257 & 0.056256\\ 0.056244 & 0 & 0.168755 & 0.056256 & 0.506245\\ 0.168744 & 0.168755 & 0 & 0.056244 & 0.056256\\ 0.506257 & 0.056256 & 0.056244 & 0 & 0.168744\\ 0.056256 & 0.506245 & 0.056256 & 0.168744 & 0 \end{array}\right)\text{ and}\\ D_{2}=\left(\begin{array}{ccccc} 0 & 0.168694 & 0.056194 & 0.506306 & 0.112556\\ 0.168694 & 0 & 0.056307 & 0.056307 & 0.562445\\ 0.056194 & 0.056307 & 0 & 0.168694 & 0.225056\\ 0.506306 & 0.056307 & 0.168694 & 0 & 0.112444\\ 0.112556 & 0.562445 & 0.225056 & 0.112444 & 0 \end{array}\right). \end{array}$$

Notice that the vector representation of *D*_1 _lies in the cone *C*_12,45 _and the vector representation of *D*_2 _lies in the cone *C*_45,12_. Thus NJ returns the same tree topology for both *D*_1 _and *D*_2_.

If we concatenate the alignments and combine the data to build one tree, then we estimate the distances using the average of *f *and *g*:

$$\frac{1}{2}(f+g)=\left(\begin{array}{ccccc} 0 & 0.102628 & 0.102624 & 0.368148 & 0.079357\\ 0.102628 & 0 & 0.102681 & 0.054222 & 0.381915\\ 0.102624 & 0.102681 & 0 & 0.102628 & 0.124313\\ 0.368148 & 0.054222 & 0.102628 & 0 & 0.127765\\ 0.079357 & 0.381915 & 0.124313 & 0.127765 & 0 \end{array}\right).$$

Using this frequency matrix we obtain the distance matrix *D*_3 _via the Jukes-Cantor correction:

$$D_{3}=\left(\begin{array}{ccccc} 0 & 0.110364 & 0.110359 & 0.506281 & 0.083878\\ 0.110364 & 0 & 0.110425 & 0.056281 & 0.533818\\ 0.110359 & 0.110425 & 0 & 0.110364 & 0.135917\\ 0.506281 & 0.056281 & 0.110364 & 0 & 0.140066\\ 0.083878 & 0.533818 & 0.135917 & 0.140066 & 0 \end{array}\right).$$

However, the vector representation of *D*_3 _lies in the cone *C*_24,15_, which means that neighbor-joining returns a different tree topology for *D*_3_. This example provides a distance-based recon-struction analog to the recent mixture model results of [14].

An analysis of the rays of $C_{n}$ suffices to prove Theorem 4.1. but the facet structure of each cone is also informative, and we were able to obtain complete information for *n *= 5. The types of facets constituting each cone are shown in Figure 1. Each cone consists of one Type A facet, two Type B facets, two Type C facets and four Type D facets. These facets intersect as follows: Type A facets are shared by pairs of cones of the form *C*_*ab*,*cd*_, *C*_*cd*,*ab*_. Type B facets are shared by pairs of cones of the form *C*_*ab*,*de*_, *C*_*ab*,*ce*_; there are two such pairs for each cone. Two of the square facets of a Type A facet belong to Type B facets, and a pair of Type B facets share a hexagon consisting of six Type I rays. The remaining two square facets of a Type A facet form Type C facets with two Type I rays. The four triangular facets of a Type A facet form Type D facets (Egyptian pyramids) with two Type I rays.

We used our description of the NJ cones to examine the *l*_2 _distance between tree metrics and the boundaries of NJ cones. Without loss of generality, by shifting the leaves in the cherries, we can assume the tree metric is of the form

$$D_{T}=\left(\begin{array}{ccccc} 0 & 0 & \alpha & \alpha +\beta & \alpha +\beta \\ 0 & 0 & \alpha & \alpha +\beta & \alpha +\beta \\ \alpha & \alpha & 0 & \beta & \beta \\ \alpha +\beta & \alpha +\beta & \beta & 0 & 0\\ \alpha +\beta & \alpha +\beta & \beta & 0 & 0 \end{array}\right)$$

where *α *and *β *are the internal branch lengths, *α *≥ $\frac{1}{2}$ and *α *+ *β *= 1. It is easy to see that *D*_*T *_∈ *C*_12,45 _confirming the consistency of neighbor-joining. The cone *C*_12,45 _contains 9 faces, but we may ignore one of them (namely the one shared with *C*_45,12_) because it is shared with a cone resulting in the same tree topology. The distance to the closest of the remaining eight faces is

(4) $$d(D_{T},{\left(C_{12,45}\cup C_{45,12}\right)}^{c})=\frac{1-\alpha }{\sqrt{3}}.$$

The *l*_2 _radius is obtained by dividing (4) by *min*(*α*, *β*), so the minimum is attained at *α *= *β *= $\frac{1}{2}$

### Theorem 4.2

*The l*_2 _*radius of neighbor-joining for *5 *taxa is *$\frac{1}{\sqrt{3}}$ ≈ 0.5773.

This is slightly larger than the *l*_∞ _radius of $\frac{1}{2}$ given by Atteson's theorem [15]. It is an interesting problem to compute the *l*_2 _radius for neighbor-joining with more taxa.

The description of the NJ cones we have provided can also be used in practice to evaluate the robustness of the algorithm when used with a specific dataset. For *n *= 5, we examined data simulated from subtrees of the two tree models *T*_1 _and *T*_2 _in [16] with the Jukes-Cantor model and the Kimura 2-parameter models [6]. For each of 40, 000 simulations, we calculated the ℓ_2_-distance between the NJ cone of the given tree and the maximum likelihood estimates for the pairwise distances (see supplementary material). These show that in many cases the maximum likelihood estimates lie very close to the boundary. In such cases, one must conclude that the NJ tree is possibly incorrect due to the variance in the distance estimates.

## 5 Optimality of the neighbor-joining algorithm

In order to study the optimality of the neighbor-joining algorithm, we compared the BME cones with the NJ cones. Such a comparison involves intersecting the cones with the ($\left(\begin{array}{c} n\\ 2 \end{array}\right)$ - 1)-sphere (in the first orthant) and then studying the volumes of their intersection by computing the standard Euclidean volume of the resulting surfaces. These surfaces are an intersection of closed hemispheres, i.e. *spherical polytopes*. Computing Euclidean volumes of (non-spherical) polytopes is a standard problem that is usually solved by triangulating and summing the volumes of the simplices. However there has been no publicly available software developed for computing or approximating volumes of spherical polytopes of dimension > 3 using this method. One possible reason for this is that in higher dimensions the volumes of spherical simplices are given by complicated analytical formulas [17] whose computational complexities are unknown.

We implemented two approaches in MATLAB (using polymake as a preprocessing step) for approximating the volume of a spherical polytope *P*. One approach is trivial: it simply samples uniformly from the sphere, and counts how many points are inside *P*. This approach is particularly suitable if *P *has large volume, or if many spherical polytopes are being simultaneously measured which partition the sphere, as is the case for NJ and BME cones. The second approach is suitable for spherical polytopes having small volume. We used this approach for computing the volumes of *consistency cones *[18] which we discuss briefly in the Discussion section.

The second approach begins by computing a triangulation of the vertices of *P *with some additional interior points of *P *added. This triangulation defines a simplicial mesh *M *which is obtained by replacing each spherical simplex with the corresponding Euclidean simplex having the same vertices. The volume of *M *(i.e. the sum of the volumes of the simplices in the mesh) is already an approximation to the volume of *P*. We refine this estimate by Monte Carlo estimation of the average value of the Jacobian from *M *to *P*. This requires sampling uniformly from *M*, which can be done very quickly in *O*(*m *+ *kd *log *d *+ *k *log *k*) time, where *m *is the number of simplices in the mesh, *k *is the number of samples, and *d *is the dimension. Briefly, the method partitions the unit interval into *m *subintervals, where the length of the *i*th subinterval is proportional to the volume of the *i*th simplex *S*_*i *_in the mesh. Then to sample *k *points from the mesh, first we decide how many of the *k *samples to draw from each *S*_*i*_, by sampling uniformly from unit interval *k *times. For each *S*_*i*_, we sample ℓ_*i *_points uniformly from *S*_*i *_where ℓ_*i *_is the number of samples *x *∈ [0, 1] which land in the *i*th subinterval. Sampling uniformly from a single simplex is a classical problem solved in *O*(*d *log *d*) time.

Our main results on the optimality of NJ for *n *= 5, 6, 7, 8 taxa are summarized in Table 3. Each row of the table describes one type of tree. Trees are classified by their topology. A *k*-cherry tree is a tree with *k *cherries. The NJ volume column shows the volume of that part of the positive orthant of dissimilarity maps for which the NJ tree is of the specified type. Similarly, the BME volume column shows the same statistic for BME trees. Finally, NJ accuracy shows the fraction of the BME cone that overlaps the NJ cone. In other words, NJ accuracy is a measure of how frequently NJ will find the BME tree for a dissimilarity map that is chosen at random.

**Table 3:** Comparison of NJ and BME cones. The volume estimates for *n *= 8 do not all add up to exactly 100% due to round-off errors

#taxa	tree shape	#trees	NJ vol	BME vol	NJ accuracy
4	unique	3	100%	100%	100%
5	unique	15	100%	100%	98.06%
6	3-cherry	15	18.49%	18.57%	90.39%
6	caterpillar	90	81.51%	81.43%	91.33%
7	3-cherry	315	45.32%	44.58%	82.42%
7	caterpillar	630	54.68%	55.42%	78.85%
8	4-cherry	315	6.48%	6.36%	70.12%
8	3-cherry (two are neighbors)	2520	27.12%	25.84%	69.93%
8	3-cherry (none are neighbors)	2520	35.67%	34.55%	71.63%
8	caterpillar	5040	30.73%	33.24%	61.75%

**Figure 4:**
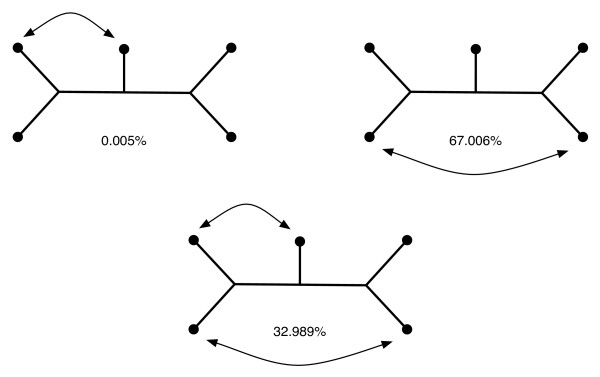
**Frequencies of the all three possible types of NJ trees that may picked instead of the BME tree for *n *= 5 leaves. **Neighbor-joining agrees with the BME tree 98.06% of the time.

We also classified and measured the intersections of NJ and BME cones in which the NJ tree differs from the BME tree. Many of these intersection cones are equivalent under the action of *S*_*n *_on the leaf labels, particularly as the stabilizer of the BME tree permutes the leaf labels in the NJ tree. In fact, for *n *= 5 taxa there are only three types of mistakes that the NJ algorithm can make when it fails to reproduce the BME tree. These are depicted in Figure 4 and the normalized spherical volumes of corresponding NJ/BME intersection cones are given.

Figure 4 can be interpreted as follows: For a random dissimilarity map, if the NJ algorithm does not produce the BME tree, then with probability 0.67 it produces the tree on the right, and if not then it almost always produces the tree in the middle. This tree differs from the BME tree significantly. A surprising result is that the tree on the left is almost *never *the NJ tree. We believe that a deeper understanding of the "mistakes" NJ makes when it does not optimize the balanced minimum evolution criterion may be important in interpreting the results, especially for large trees.

We also computed analogous results for *n *= 6, 7, 8, 9, 10. They are available, together with the software for computing volumes at [19].

## 6 Discussion

Theoretical studies of the neighbor-joining algorithm have focused on statistical consistency and the robustness of the algorithm to small perturbations of tree metrics. The paper by [20] established the consistency of NJ, that is, if *D*_*T *_is a tree metric then NJ outputs the tree *T*. This result was then extended in [15] and more recently by [18] who show that if *D *is "close" to a tree metric *D*_*T *_for some *T*, then NJ outputs *T *on input *D*.

Our results provide a different perspective on the NJ algorithm. Namely, we address the question of the accuracy of the greedy approach for the underlying linear programming problem of BME optimization. This led us to the study of BME polytopes, and the combinatorics of these polytopes is interesting in its own right:

### Question 6.1

*Is there a combinatorial criterion for two tree topologies forming an edge in the BME polytope, similar to pruning/re-grafting or some other operation on trees? If so, this could be used to define a combinatorial pivoting rule on tree space that could be used in hill-climbing algorithms for phylogenetic reconstruction. Such a pivoting rule would have the advantage that it would be equivalent to performing an edge-walk on the BME polytope. Edge-walking methods are known to perform well in practice for solving linear programs. See *[21]*for an example of a local search approach to finding minimum evolution trees*.

Similarly, a better understanding of the combinatorics of the NJ cones will lead to a clearer view of the strengths and weaknesses of the neighbor-joining algorithm. A basic problem is the following:

### Question 6.2

Find a combinatorial description of the NJ cones for general n. How many facets/rays are there?

Our computational results lend new insights into the performance of the NJ and BME algorithms for small trees. We have measured the relative sizes of cones for different shapes of trees, and measured the frequencies of all combinatorial types of discrepancies between BME and NJ trees. In particular, we have observed that the NJ algorithm is least likely to reproduce the BME tree when the BME tree is the caterpillar tree.

### Conjecture 6.3

*For n *> 6, *it is the caterpillar tree that yields the smallest ratio of spherical cone volumes vol(NJ ⋂ BME)/vol(BME) where NJ is the spherical cone volume of a union of the NJ cones and BME is the spherical cone volume of the BME cone for a fixed tree. In other words, the caterpillar tree is the most difficult BME tree topology for the NJ algorithm to reproduce*.

Another problem we believe is very important is to extend the results shown in Figure 4 to large trees. In other words, to understand how neighbor-joining can fail when it does not succeed in finding the balanced minimum evolution tree.

### Question 6.4

What tree topologies is neighbor-joining likely to pick when it fails to construct the balanced minimum evolution tree?

There are many other interesting cones related to distance-based methods that can be considered in this context. For example, in [18], it is shown that the *quartet consistency *condition is sufficient for neighbor-joining to reconstruct a tree from a dissimilarity map for *n *≤ 7 leaves. The quartet consistency conditions define polyhedral cones (consistency cones) in $R^{\left(\begin{array}{c} n\\ 2 \end{array}\right)}$; see [18] for details. For *n *= 4 taxa the consistency cones cover all of $R^{\left(\begin{array}{c} 4\\ 2 \end{array}\right)}$ showing that quartet consistency explains the behavior of neighbor-joining for all dissimilarity maps. Using the second method outlined in Section 4 we succeeded in computing the volumes of the consistency cones intersected with the first orthant of the sphere for *n *= 5 taxa. There are 15 cones, all equivalent under orthogonal transformation, and their union covers 27.93% of $R_{+}^{\left(\begin{array}{c} 5\\ 2 \end{array}\right)}$, measured with respect to spherical volume. In other words, quartet consistency explains the behavior of neighbor-joining on almost $\frac{1}{3}$ of dissimilarity maps.

Such computations are pushing the boundary of computational polyhedral geometry. For *n *≥ 6 taxa, triangulating a consistency cone is too unwieldy, although we are confident that spherical volumes could still be computed using polynomial time hit-and-run sampling methods for volume approximation [22]. Such methods are complicated and not yet implemented.

Finally, we comment on the example in Section 3 that shows how different alignments may lead to the same neighbor-joining tree, whereas the neighbor-joining tree constructed from a concatenation of the alignments is different. This result has significant implications for studies where species trees are constructed from multiple gene families by combining the data.

## 7 Competing interests

The authors declare that they have no competing interests.
